# *N*,*N*′-Dibenzyl-*N*′′-(2-chloro-2,2-difluoro­acet­yl)-*N*,*N*′-dimethyl­phospho­ric triamide

**DOI:** 10.1107/S1600536811005423

**Published:** 2011-02-19

**Authors:** Akbar Raissi Shabari, Mehrdad Pourayoubi, Anahid Saneei

**Affiliations:** a Faculty of Chemistry, Islamic Azad University, North Tehran Branch, Tehran, Iran; b Department of Chemistry, Ferdowsi University of Mashhad, Mashhad 91779, Iran

## Abstract

In the title mol­ecule, C_18_H_21_ClF_2_N_3_O_2_P, the P=O and N—H groups are *syn* to each other. The P atom adopts a slightly distorted tetra­hedral environment and the N atoms of the tertiary amine groups are bonded in an essentially planar geometry. In the crystal, pairs of inter­molecular N—H⋯O(P) hydrogen bonds form centrosymmetric dimers.

## Related literature

For metal complexes with phosphoryl donor ligands, see: Gholivand *et al.* (2010 ▶). For a polyoxometalate-based inorganic–organic compound containing a phosphoryl ligand, see: Niu *et al.* (1996 ▶). For phospho­ric triamide compounds having a C(=O)NHP(=O) skeleton, see: Pourayoubi & Sabbaghi (2009 ▶) and references cited therein. For bond lengths in related structures, see: Sabbaghi *et al.* (2010 ▶). For hydrogen-bond motifs, see: Etter *et al.* (1990 ▶); Bernstein *et al.* (1995 ▶). For the synthesis of the starting material, CClF_2_C(O)NHP(O)Cl_2_, see: Iriarte *et al.* (2008 ▶).
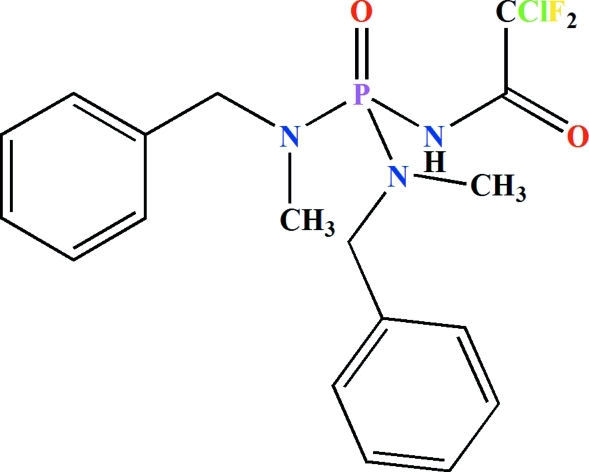


## Experimental

### 

#### Crystal data

C_18_H_21_ClF_2_N_3_O_2_P
*M* *_r_* = 415.80
Triclinic, 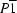

*a* = 10.3059 (9) Å
*b* = 10.5030 (9) Å
*c* = 10.9473 (9) Å
α = 71.743 (2)°
β = 67.294 (2)°
γ = 63.265 (2)°
*V* = 962.15 (14) Å^3^
*Z* = 2
Mo *K*α radiation
μ = 0.32 mm^−1^
*T* = 120 K
0.28 × 0.22 × 0.15 mm

#### Data collection

Bruker SMART 1000 CCD area-detector diffractometer
Absorption correction: multi-scan (*SADABS*; Sheldrick, 1998 ▶) *T*_min_ = 0.916, *T*_max_ = 0.954
9258 measured reflections
4148 independent reflections
3359 reflections with *I* > 2σ(*I*)
*R*_int_ = 0.025

#### Refinement

*R*[*F* ^2^ > 2σ(*F* ^2^)] = 0.049
*wR*(*F* ^2^) = 0.111
*S* = 1.00
4148 reflections
247 parameters
H-atom parameters constrained
Δρ_max_ = 0.39 e Å^−3^
Δρ_min_ = −0.37 e Å^−3^

### 

Data collection: *SMART* (Bruker, 1998 ▶); cell refinement: *SAINT-Plus* (Bruker, 1998 ▶); data reduction: *SAINT-Plus*; program(s) used to solve structure: *SHELXTL* (Sheldrick, 2008 ▶); program(s) used to refine structure: *SHELXTL*; molecular graphics: *SHELXTL*; software used to prepare material for publication: *SHELXTL*.

## Supplementary Material

Crystal structure: contains datablocks I, global. DOI: 10.1107/S1600536811005423/lh5205sup1.cif

Structure factors: contains datablocks I. DOI: 10.1107/S1600536811005423/lh5205Isup2.hkl

Additional supplementary materials:  crystallographic information; 3D view; checkCIF report

## Figures and Tables

**Table 1:** Selected bond angles (°)

O1—P1—N2	112.34 (9)
O1—P1—N1	117.13 (9)
N2—P1—N1	107.02 (9)
O1—P1—N3	105.06 (9)
N2—P1—N3	110.57 (9)
N1—P1—N3	104.38 (9)
C1—N1—C2	114.36 (17)
C1—N1—P1	126.21 (15)
C2—N1—P1	119.42 (14)
C9—N2—C10	115.45 (17)
C9—N2—P1	121.56 (14)
C10—N2—P1	122.42 (15)

**Table 2:** Hydrogen-bond geometry (Å, °)

*D*—H⋯*A*	*D*—H	H⋯*A*	*D*⋯*A*	*D*—H⋯*A*
N3—H3*N*⋯O1^i^	0.87	1.91	2.772 (3)	174

Symmetry code: (i) 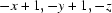.

**Fig. 1.**
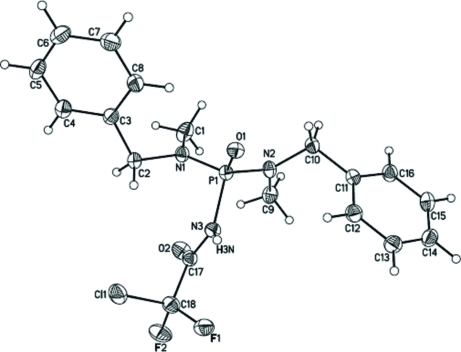
The molecular structure of the title compound with displacement ellipsoids at the 50% probability level.

**Fig. 2.**
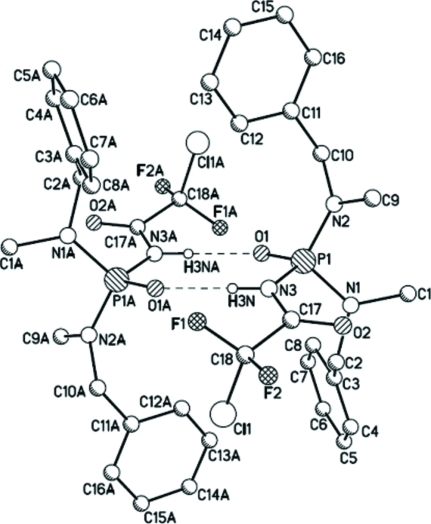
A view of the H-bonded (dashed lines) centrosymmetric dimer (symmetry code: (A) 1-x, 1-y, -z).

## supplementary crystallographic information

### Comment

Phosphoryl donor ligands have been used to prepare coordination complexes (Gholivand *et al.*, 2010) and polyoxometalate-based inorganic-organic hybrid compounds (Niu *et al.*, 1996). The structure determination of title compound was performed as a part of a project on the synthesis of new potential phosphoric triamide ligands having a C(═O)NHP(═O) skeleton (Pourayoubi & Sabbaghi, 2009).

In the title compound, C_18_H_21_ClF_2_N_3_O_2_P, the phosphoryl group and NH unit are *syn* to each other and the phosphorus atom has a slightly distorted tetrahedral configuration (Fig. 1). The P atom adopts a slightly distorted tetrahedral environment and the N atoms of the tertiary amine groups are bonded in an essentially planar geometry (see Table 1). The P═O bond length is comparable to those in similar compounds e.g. in P(O)[NHC(O)C_6_H_4_(4-NO_2_)][NHC_6_H_11_]_2_ (Sabbaghi *et al.*, 2010). In the (CClF_2_)C(O) unit, the O—C—C—F dihedral angles showing the orientation of flourine atoms relative to carbonyl group are 17.7 (3) and 137.1 (2)° and the O—C—C—Cl dihedral angle is -101.9 (2)°.

The phosphoryl is a better H-acceptor relative to the carbonyl counterpart (Pourayoubi & Sabbaghi, 2009); so, the hydrogen atom of the C(═ O)NHP(═ O) group is involved in an intermolecular –P═O···H—N– hydrogen bond (see Table 2) to form a centrosymmetric dimeric aggregate [graph set: *R*_2_^2^(8) (Etter *et al.*, 1990; Bernstein *et al.*, 1995)], Fig. 2.

### Experimental

Synthesis of CClF_2_C(O)NHP(O)Cl_2_: CClF_2_C(O)NHP(O)Cl_2_ was prepared according to procedure reported by Iriarte *et al.* (2008) from a reaction between phosphorus pentachloride (16.91 mmol) and CClF_2_C(O)NH_2_ (16.91 mmol) in dry CCl_4_ at 358 K (3 h) and then the treatment of formic acid (16.91 mmol) at ice bath temperature; then removing of solvent in vacuum to yield CClF_2_C(O)NHP(O)Cl_2_.

Synthesis of title compound: To a solution of CClF_2_C(O)NHP(O)Cl_2_ (1.04 mmol) in dry CHCl_3_, a solution of *N*-methylbenzylamine (4.16 mmol) in dry CHCl_3_ was added dropwise and stirred at 273 K. After 4 h, the solvent was removed at room temperature. The solid was washed with H_2_O. The product was obtained after recrystallization from a methanol/n-heptane mixture (4:1) after a slow evaporation at room temperature. IR (KBr, cm^-1^): 3066, 2886, 1729 (C═O), 1592, 1481, 1359, 1286, 1218, 1150, 1009, 965, 863, 809, 698. ^19^F NMR (470.59 MHz, DMSO-d6, 300 K, CFCl_3_): -63.69 p.p.m. (*s*). ^31^P{^1^H} NMR (202.46 MHz, DMSO-d6, 300 K, 85% H_3_PO_4_): 12.80 p.p.m. (*s*). ^1^H NMR (500.13 MHz, DMSO-d6, 300 K, TMS): 2.48 (*s*, 3H, CH_3_), 2.50 (*s*, 3H, CH_3_), 4.16 (*m*, 4H, CH_2_), 7.25–7.38 (*m*, 10H, Ar–H), 10.60 p.p.m. (*s*, 1H, NH). ^13^C NMR (125.76 MHz, DMSO-d6, 300 K, TMS): 33.16 (*d*, ^2^J(P,*C*) = 4.6 Hz, 2 C, CH_3_), 51.78 (*d*, ^2^J(P,*C*) = 5.1 Hz, 2 C, CH_2_), 118.08 (*dt*, 1C, CClF_2_), 127.17 (*s*), 127.96 (*s*), 128.32 (*s*), 137.68 (*d*, ^3^J(P,*C*) = 3.9 Hz, 2 C, C*_ipso_*), 159.81 p.p.m. (*t*, ^2^J(F,*C*) = 35.0 Hz, 1 C, C═O).

### Refinement

H atoms were placed in calculated postions with C—H = 0.95 - 0.99Å and N—H = 0.87Å and were included in the refinement with U_iso_(H) = 1.2U_eq_(C) or 1.5U_eq_(C_methyl_). The U_iso_(H) value of the H atom bonded to N3 was refined.

### Crystal data

**Table:**

C_18_H_21_ClF_2_N_3_O_2_P	*Z* = 2
*M**_r_* = 415.80	*F*(000) = 432
Triclinic, *P*1	*D*_x_ = 1.435 Mg m^−^^3^
Hall symbol: -P 1	Mo *K*α radiation, λ = 0.71073 Å
*a* = 10.3059 (9) Å	Cell parameters from 3946 reflections
*b* = 10.5030 (9) Å	θ = 2.3–29.1°
*c* = 10.9473 (9) Å	µ = 0.32 mm^−^^1^
α = 71.743 (2)°	*T* = 120 K
β = 67.294 (2)°	Prism, colourless
γ = 63.265 (2)°	0.28 × 0.22 × 0.15 mm
*V* = 962.15 (14) Å^3^	

### Data collection

**Table:**

Bruker SMART 1000 CCD area-detector diffractometer	4148 independent reflections
Radiation source: fine-focus sealed tube	3359 reflections with *I* > 2σ(*I*)
graphite	*R*_int_ = 0.025
φ and ω scans	θ_max_ = 27.0°, θ_min_ = 2.1°
Absorption correction: multi-scan (*SADABS*; Sheldrick, 1998)	*h* = −13→13
*T*_min_ = 0.916, *T*_max_ = 0.954	*k* = −13→13
9258 measured reflections	*l* = −13→13

### Refinement

**Table:**

Refinement on *F*^2^	Primary atom site location: structure-invariant direct methods
Least-squares matrix: full	Secondary atom site location: difference Fourier map
*R*[*F*^2^ > 2σ(*F*^2^)] = 0.049	Hydrogen site location: mixed
*wR*(*F*^2^) = 0.111	H-atom parameters constrained
*S* = 1.00	*w* = 1/[σ^2^(*F*_o_^2^) + (0.0309*P*)^2^ + 1.6759*P*] where *P* = (*F*_o_^2^ + 2*F*_c_^2^)/3
4148 reflections	(Δ/σ)_max_ < 0.001
247 parameters	Δρ_max_ = 0.39 e Å^−^^3^
0 restraints	Δρ_min_ = −0.37 e Å^−^^3^

### Special details

**Table:**

Geometry. All e.s.d.'s (except the e.s.d. in the dihedral angle between two l.s. planes) are estimated using the full covariance matrix. The cell e.s.d.'s are taken into account individually in the estimation of e.s.d.'s in distances, angles and torsion angles; correlations between e.s.d.'s in cell parameters are only used when they are defined by crystal symmetry. An approximate (isotropic) treatment of cell e.s.d.'s is used for estimating e.s.d.'s involving l.s. planes.
Refinement. Refinement of *F*^2^ against ALL reflections. The weighted *R*-factor *wR* and goodness of fit *S* are based on *F*^2^, conventional *R*-factors *R* are based on *F*, with *F* set to zero for negative *F*^2^. The threshold expression of *F*^2^ > σ(*F*^2^) is used only for calculating *R*-factors(gt) *etc*. and is not relevant to the choice of reflections for refinement. *R*-factors based on *F*^2^ are statistically about twice as large as those based on *F*, and *R*- factors based on ALL data will be even larger.

### Fractional atomic coordinates and isotropic or equivalent isotropic displacement parameters (Å^2^)

**Table:**

	*x*	*y*	*z*	*U*_iso_*/*U*_eq_	
P1	0.24133 (6)	0.55399 (6)	0.07097 (5)	0.01766 (13)	
Cl1	0.40224 (7)	0.93801 (7)	−0.06620 (6)	0.03255 (16)	
F1	0.45008 (15)	0.79512 (15)	0.15810 (14)	0.0304 (3)	
F2	0.25207 (16)	0.99322 (15)	0.16926 (15)	0.0346 (3)	
O1	0.37544 (16)	0.44767 (15)	−0.00684 (15)	0.0207 (3)	
O2	0.09472 (17)	0.85802 (17)	0.16326 (17)	0.0290 (4)	
N1	0.11179 (19)	0.66717 (19)	−0.00324 (17)	0.0194 (4)	
N2	0.1560 (2)	0.47741 (19)	0.21311 (17)	0.0205 (4)	
N3	0.3102 (2)	0.66075 (19)	0.09736 (18)	0.0197 (4)	
H3N	0.4088	0.6297	0.0731	0.039 (8)*	
C1	−0.0432 (3)	0.6755 (3)	0.0358 (2)	0.0300 (5)	
H1A	−0.0678	0.6791	−0.0435	0.045*	
H1B	−0.1129	0.7630	0.0751	0.045*	
H1C	−0.0534	0.5902	0.1021	0.045*	
C2	0.1497 (3)	0.7723 (2)	−0.1223 (2)	0.0232 (4)	
H2A	0.2436	0.7795	−0.1257	0.028*	
H2B	0.0671	0.8685	−0.1125	0.028*	
C3	0.1726 (2)	0.7347 (2)	−0.2540 (2)	0.0215 (4)	
C4	0.1249 (2)	0.8454 (2)	−0.3561 (2)	0.0241 (5)	
H4A	0.0693	0.9420	−0.3396	0.029*	
C5	0.1583 (3)	0.8152 (3)	−0.4825 (2)	0.0281 (5)	
H5A	0.1265	0.8912	−0.5521	0.034*	
C6	0.2378 (3)	0.6744 (3)	−0.5067 (2)	0.0299 (5)	
H6A	0.2612	0.6537	−0.5929	0.036*	
C7	0.2835 (3)	0.5633 (3)	−0.4041 (2)	0.0293 (5)	
H7A	0.3371	0.4666	−0.4204	0.035*	
C8	0.2511 (3)	0.5929 (2)	−0.2784 (2)	0.0254 (5)	
H8A	0.2825	0.5165	−0.2088	0.030*	
C9	0.0398 (3)	0.5614 (2)	0.3182 (2)	0.0254 (5)	
H9A	−0.0450	0.5282	0.3573	0.038*	
H9B	0.0031	0.6641	0.2790	0.038*	
H9C	0.0839	0.5478	0.3885	0.038*	
C10	0.2044 (3)	0.3195 (2)	0.2526 (2)	0.0235 (4)	
H10A	0.2779	0.2738	0.1736	0.028*	
H10B	0.1151	0.2927	0.2808	0.028*	
C11	0.2766 (2)	0.2604 (2)	0.3657 (2)	0.0217 (4)	
C12	0.4040 (3)	0.2854 (2)	0.3528 (2)	0.0249 (5)	
H12A	0.4465	0.3391	0.2720	0.030*	
C13	0.4683 (3)	0.2321 (2)	0.4577 (2)	0.0263 (5)	
H13A	0.5547	0.2497	0.4485	0.032*	
C14	0.4077 (3)	0.1535 (2)	0.5757 (2)	0.0267 (5)	
H14A	0.4521	0.1177	0.6473	0.032*	
C15	0.2827 (3)	0.1271 (2)	0.5896 (2)	0.0279 (5)	
H15A	0.2415	0.0723	0.6702	0.034*	
C16	0.2175 (3)	0.1812 (2)	0.4847 (2)	0.0250 (5)	
H16A	0.1309	0.1635	0.4948	0.030*	
C17	0.2317 (2)	0.7958 (2)	0.1275 (2)	0.0213 (4)	
C18	0.3323 (3)	0.8782 (2)	0.1073 (2)	0.0242 (5)	

### Atomic displacement parameters (Å^2^)

**Table:**

	*U*^11^	*U*^22^	*U*^33^	*U*^12^	*U*^13^	*U*^23^
P1	0.0166 (3)	0.0174 (3)	0.0179 (3)	−0.0063 (2)	−0.0047 (2)	−0.0021 (2)
Cl1	0.0383 (3)	0.0335 (3)	0.0279 (3)	−0.0217 (3)	−0.0053 (2)	−0.0007 (2)
F1	0.0315 (7)	0.0306 (7)	0.0366 (8)	−0.0135 (6)	−0.0154 (6)	−0.0052 (6)
F2	0.0362 (8)	0.0289 (7)	0.0408 (8)	−0.0132 (6)	−0.0024 (6)	−0.0182 (6)
O1	0.0184 (7)	0.0203 (7)	0.0230 (8)	−0.0061 (6)	−0.0055 (6)	−0.0059 (6)
O2	0.0209 (8)	0.0242 (8)	0.0371 (9)	−0.0052 (7)	−0.0030 (7)	−0.0108 (7)
N1	0.0179 (8)	0.0201 (9)	0.0189 (9)	−0.0076 (7)	−0.0057 (7)	−0.0004 (7)
N2	0.0197 (9)	0.0193 (9)	0.0186 (8)	−0.0077 (7)	−0.0038 (7)	0.0002 (7)
N3	0.0167 (8)	0.0188 (9)	0.0237 (9)	−0.0056 (7)	−0.0065 (7)	−0.0044 (7)
C1	0.0195 (11)	0.0406 (14)	0.0272 (12)	−0.0104 (10)	−0.0092 (9)	−0.0002 (10)
C2	0.0294 (11)	0.0194 (10)	0.0213 (11)	−0.0098 (9)	−0.0103 (9)	0.0008 (8)
C3	0.0211 (10)	0.0259 (11)	0.0195 (10)	−0.0124 (9)	−0.0057 (8)	−0.0013 (8)
C4	0.0208 (10)	0.0270 (11)	0.0239 (11)	−0.0105 (9)	−0.0070 (9)	−0.0005 (9)
C5	0.0277 (12)	0.0373 (13)	0.0210 (11)	−0.0146 (10)	−0.0114 (9)	0.0023 (9)
C6	0.0314 (12)	0.0449 (14)	0.0208 (11)	−0.0212 (11)	−0.0062 (9)	−0.0060 (10)
C7	0.0321 (12)	0.0300 (12)	0.0285 (12)	−0.0155 (10)	−0.0046 (10)	−0.0079 (10)
C8	0.0282 (11)	0.0242 (11)	0.0246 (11)	−0.0125 (9)	−0.0084 (9)	0.0001 (9)
C9	0.0250 (11)	0.0295 (12)	0.0173 (10)	−0.0114 (9)	−0.0016 (9)	−0.0023 (9)
C10	0.0275 (11)	0.0203 (10)	0.0243 (11)	−0.0116 (9)	−0.0102 (9)	0.0016 (8)
C11	0.0236 (10)	0.0171 (10)	0.0237 (11)	−0.0063 (8)	−0.0084 (9)	−0.0025 (8)
C12	0.0253 (11)	0.0253 (11)	0.0236 (11)	−0.0119 (9)	−0.0055 (9)	−0.0020 (9)
C13	0.0222 (11)	0.0258 (11)	0.0317 (12)	−0.0078 (9)	−0.0096 (9)	−0.0054 (9)
C14	0.0273 (11)	0.0249 (11)	0.0264 (11)	−0.0042 (9)	−0.0125 (9)	−0.0051 (9)
C15	0.0308 (12)	0.0236 (11)	0.0241 (11)	−0.0102 (10)	−0.0080 (9)	0.0026 (9)
C16	0.0242 (11)	0.0245 (11)	0.0268 (11)	−0.0116 (9)	−0.0092 (9)	0.0013 (9)
C17	0.0235 (11)	0.0205 (10)	0.0192 (10)	−0.0088 (9)	−0.0054 (8)	−0.0025 (8)
C18	0.0249 (11)	0.0205 (10)	0.0261 (11)	−0.0072 (9)	−0.0055 (9)	−0.0068 (9)

### Geometric parameters (Å, °)

**Table:**

P1—O1	1.4769 (15)	C5—H5A	0.9500
P1—N2	1.6241 (18)	C6—C7	1.391 (3)
P1—N1	1.6308 (18)	C6—H6A	0.9500
P1—N3	1.7093 (18)	C7—C8	1.387 (3)
Cl1—C18	1.768 (2)	C7—H7A	0.9500
F1—C18	1.345 (3)	C8—H8A	0.9500
F2—C18	1.336 (2)	C9—H9A	0.9800
O2—C17	1.215 (3)	C9—H9B	0.9800
N1—C1	1.453 (3)	C9—H9C	0.9800
N1—C2	1.471 (3)	C10—C11	1.512 (3)
N2—C9	1.469 (3)	C10—H10A	0.9900
N2—C10	1.469 (3)	C10—H10B	0.9900
N3—C17	1.353 (3)	C11—C16	1.387 (3)
N3—H3N	0.8700	C11—C12	1.399 (3)
C1—H1A	0.9800	C12—C13	1.387 (3)
C1—H1B	0.9800	C12—H12A	0.9500
C1—H1C	0.9800	C13—C14	1.383 (3)
C2—C3	1.517 (3)	C13—H13A	0.9500
C2—H2A	0.9900	C14—C15	1.379 (3)
C2—H2B	0.9900	C14—H14A	0.9500
C3—C4	1.393 (3)	C15—C16	1.391 (3)
C3—C8	1.394 (3)	C15—H15A	0.9500
C4—C5	1.394 (3)	C16—H16A	0.9500
C4—H4A	0.9500	C17—C18	1.543 (3)
C5—C6	1.384 (4)		
			
O1—P1—N2	112.34 (9)	C7—C8—C3	120.0 (2)
O1—P1—N1	117.13 (9)	C7—C8—H8A	120.0
N2—P1—N1	107.02 (9)	C3—C8—H8A	120.0
O1—P1—N3	105.06 (9)	N2—C9—H9A	109.5
N2—P1—N3	110.57 (9)	N2—C9—H9B	109.5
N1—P1—N3	104.38 (9)	H9A—C9—H9B	109.5
C1—N1—C2	114.36 (17)	N2—C9—H9C	109.5
C1—N1—P1	126.21 (15)	H9A—C9—H9C	109.5
C2—N1—P1	119.42 (14)	H9B—C9—H9C	109.5
C9—N2—C10	115.45 (17)	N2—C10—C11	113.28 (18)
C9—N2—P1	121.56 (14)	N2—C10—H10A	108.9
C10—N2—P1	122.42 (15)	C11—C10—H10A	108.9
C17—N3—P1	127.29 (15)	N2—C10—H10B	108.9
C17—N3—H3N	117.5	C11—C10—H10B	108.9
P1—N3—H3N	114.0	H10A—C10—H10B	107.7
N1—C1—H1A	109.5	C16—C11—C12	118.6 (2)
N1—C1—H1B	109.5	C16—C11—C10	120.9 (2)
H1A—C1—H1B	109.5	C12—C11—C10	120.59 (19)
N1—C1—H1C	109.5	C13—C12—C11	120.2 (2)
H1A—C1—H1C	109.5	C13—C12—H12A	119.9
H1B—C1—H1C	109.5	C11—C12—H12A	119.9
N1—C2—C3	114.05 (17)	C14—C13—C12	120.4 (2)
N1—C2—H2A	108.7	C14—C13—H13A	119.8
C3—C2—H2A	108.7	C12—C13—H13A	119.8
N1—C2—H2B	108.7	C15—C14—C13	120.1 (2)
C3—C2—H2B	108.7	C15—C14—H14A	120.0
H2A—C2—H2B	107.6	C13—C14—H14A	120.0
C4—C3—C8	119.4 (2)	C14—C15—C16	119.6 (2)
C4—C3—C2	119.3 (2)	C14—C15—H15A	120.2
C8—C3—C2	121.15 (19)	C16—C15—H15A	120.2
C3—C4—C5	120.3 (2)	C11—C16—C15	121.2 (2)
C3—C4—H4A	119.8	C11—C16—H16A	119.4
C5—C4—H4A	119.8	C15—C16—H16A	119.4
C6—C5—C4	120.1 (2)	O2—C17—N3	127.4 (2)
C6—C5—H5A	120.0	O2—C17—C18	118.39 (19)
C4—C5—H5A	120.0	N3—C17—C18	114.16 (18)
C5—C6—C7	119.7 (2)	F2—C18—F1	107.18 (18)
C5—C6—H6A	120.1	F2—C18—C17	110.49 (18)
C7—C6—H6A	120.1	F1—C18—C17	111.98 (17)
C8—C7—C6	120.5 (2)	F2—C18—Cl1	108.70 (15)
C8—C7—H7A	119.8	F1—C18—Cl1	109.06 (15)
C6—C7—H7A	119.8	C17—C18—Cl1	109.35 (15)
			
O1—P1—N1—C1	115.12 (19)	C6—C7—C8—C3	−0.1 (3)
N2—P1—N1—C1	−12.0 (2)	C4—C3—C8—C7	1.2 (3)
N3—P1—N1—C1	−129.24 (19)	C2—C3—C8—C7	−173.7 (2)
O1—P1—N1—C2	−64.27 (18)	C9—N2—C10—C11	−61.8 (2)
N2—P1—N1—C2	168.61 (15)	P1—N2—C10—C11	109.64 (19)
N3—P1—N1—C2	51.38 (17)	N2—C10—C11—C16	122.1 (2)
O1—P1—N2—C9	167.42 (16)	N2—C10—C11—C12	−57.4 (3)
N1—P1—N2—C9	−62.69 (18)	C16—C11—C12—C13	−0.2 (3)
N3—P1—N2—C9	50.40 (19)	C10—C11—C12—C13	179.2 (2)
O1—P1—N2—C10	−3.55 (19)	C11—C12—C13—C14	0.2 (3)
N1—P1—N2—C10	126.34 (17)	C12—C13—C14—C15	0.3 (3)
N3—P1—N2—C10	−120.57 (16)	C13—C14—C15—C16	−0.6 (3)
O1—P1—N3—C17	158.89 (18)	C12—C11—C16—C15	−0.1 (3)
N2—P1—N3—C17	−79.68 (19)	C10—C11—C16—C15	−179.6 (2)
N1—P1—N3—C17	35.1 (2)	C14—C15—C16—C11	0.6 (4)
C1—N1—C2—C3	−75.1 (2)	P1—N3—C17—O2	13.3 (3)
P1—N1—C2—C3	104.32 (19)	P1—N3—C17—C18	−164.20 (15)
N1—C2—C3—C4	143.87 (19)	O2—C17—C18—F2	17.7 (3)
N1—C2—C3—C8	−41.2 (3)	N3—C17—C18—F2	−164.59 (18)
C8—C3—C4—C5	−1.5 (3)	O2—C17—C18—F1	137.1 (2)
C2—C3—C4—C5	173.5 (2)	N3—C17—C18—F1	−45.2 (2)
C3—C4—C5—C6	0.7 (3)	O2—C17—C18—Cl1	−101.9 (2)
C4—C5—C6—C7	0.4 (3)	N3—C17—C18—Cl1	75.8 (2)
C5—C6—C7—C8	−0.6 (4)		

### Hydrogen-bond geometry (Å, °)

**Table:**

*D*—H···*A*	*D*—H	H···*A*	*D*···*A*	*D*—H···*A*
N3—H3N···O1^i^	0.87	1.91	2.772 (3)	174

Symmetry codes: (i) −*x*+1, −*y*+1, −*z*.
